# Applications of SPECT and PET Imaging for the Physiological Evaluation of Lower Extremity Peripheral Artery Disease

**DOI:** 10.3390/ijms25137474

**Published:** 2024-07-08

**Authors:** Eleanor T. Rimmerman, Mitchel R. Stacy

**Affiliations:** 1 Interdisciplinary Biophysics Graduate Program, The Ohio State University, Columbus, OH 43210, USA; 2 Center for Regenerative Medicine, Research Institute at Nationwide Children’s Hospital, Columbus, OH 43215, USA; 3 Division of Vascular Diseases and Surgery, Department of Surgery, The Ohio State University College of Medicine, Columbus, OH 43210, USA

**Keywords:** peripheral artery disease, chronic limb-threatening ischemia, atherosclerosis, perfusion, imaging, nuclear medicine, SPECT, PET

## Abstract

Peripheral artery disease (PAD) is classified as the narrowing or complete occlusion of the lower extremity arteries due to atherosclerosis. The risk of developing PAD increases with increased age and risk factors such as smoking, diabetes, hypertension, and hypercholesterolemia. Current treatment for PAD involves lifestyle and symptom management, statin and antiplatelet therapy, and/or surgical interventions to improve quality of life with varying efficacy. PAD affects approximately 5 to 6 percent of the global population, with this global burden continuing to increase. Despite the increase in disease prevalence, no gold standard functional diagnostic tool has been established for enabling early detection of the disease, appropriate medical management, and prediction of adverse outcomes for PAD patients. The visualization and quantification of the physiological consequences of PAD are possible by way of nuclear imaging: specifically, via scintigraphy, single-photon emission computed tomography (SPECT), and positron emission tomography (PET) imaging. These non-invasive modalities, when combined with targeted radionuclides, possess utility for detecting functional perfusion deficits and provide unique insight into muscle tissue- and vascular-level characteristics of PAD patients. This review discusses the past, present, and emerging applications of hybrid nuclear imaging modalities in the evaluation and monitoring of patients with PAD.

## 1. Introduction

Peripheral artery disease (PAD) is a progressive atherosclerotic disorder characterized by arterial stenosis and plaque deposition in the lower extremities. Left untreated or poorly managed, PAD can lead to lower extremity claudication, non-healing ulcers of the ankles and feet, gangrene, amputation, cerebrovascular events, myocardial infarction, and death [1]. First-line treatment involves lifestyle modifications (diet, smoking cessation [2], and high-intensity exercise [3]) in combination with statin and antiplatelet therapy aimed at stabilizing atherosclerotic plaques, lowering low-density lipoprotein cholesterol, and improving symptoms [4]. More aggressive methods include invasive surgical interventions such as endovascular procedures (balloon angioplasty, stenting) or bypass grafting [5]. Despite these treatment efforts, the global incidence of peripheral artery disease has increased from approximately 65.8 million in 1990 to 113 million in 2019 [6]. Current screening tools commonly focus on identifying lower extremity PAD through the assessment of hemodynamics (e.g., ankle-brachial index, toe-brachial index) or vascular anatomy (e.g., ultrasound, angiography). Therefore, additional development of non-invasive diagnostic approaches that allow for combined monitoring of both functional and anatomical consequences of PAD could potentially improve the standard of care for disease detection, management, and monitoring.

Since the early 1970s, nuclear medicine strategies have evolved for evaluating cardiovascular diseases, with primary efforts focusing on the assessment of myocardial perfusion deficits in patients with coronary artery disease [7]. However, in recent decades, single-photon emission computed tomography (SPECT) and positron emission tomography (PET), when partnered with targeted radionuclides, have become increasingly recognized as non-invasive imaging approaches that may also identify abnormalities in lower extremity skeletal muscle perfusion, localized regions of soft tissue and bone infection, and peripheral atherosclerosis in patients with PAD (Table 1).

This review briefly discusses the history of non-invasive assessment of clinical PAD with nuclear imaging techniques, highlights emerging developments and applications of SPECT and PET imaging in patients with PAD, and discusses potential future directions and roles of nuclear imaging in the early detection and management of PAD.

## 2. SPECT/CT Imaging

In the 1940s, the first nuclear medicine investigations to evaluate lower extremity blood flow in patients with PAD used a Geiger meter to track dynamic changes in radioactive counts following the administration of radioisotopes [52,53]. The creation and evolution of non-invasive nuclear cardiology then subsequently led to the application of two-dimensional scintigraphy in PAD patients for the analysis of limb perfusion [8,10,54]. However, it was not until 1989 that SPECT imaging was first utilized for the three-dimensional (3D) assessment of relative perfusion in PAD patients [10]. Thallium-201 (^201^Tl) became the first radionuclide of choice for early SPECT imaging investigations focused on evaluating perfusion in PAD, with these studies demonstrating the ability to non-invasively detect perfusion deficits in the anterior and posterior compartments of the lower extremities [10]. However, relative limitations of ^201^Tl, including a 73 h half-life and a relatively low energy emission of 78 keV for gamma imaging, led to the utilization of radionuclides that possessed more favorable imaging attributes [1]. Most notably, from the 1990s onward, technetium-99m (^99m^Tc)-labeled radionuclides, including ^99m^Tc-sestamibi and ^99m^Tc-tetrofosmin, became the most widely used for SPECT imaging investigations in clinical PAD due to their more favorable gamma imaging properties (i.e., 6 h half-life and 140 keV emission of ^99m^Tc), which reduced the amount of ionizing radiation for patients while simultaneously improving image quality.

### 2.1. Skeletal Muscle Perfusion

**Figure 1:**
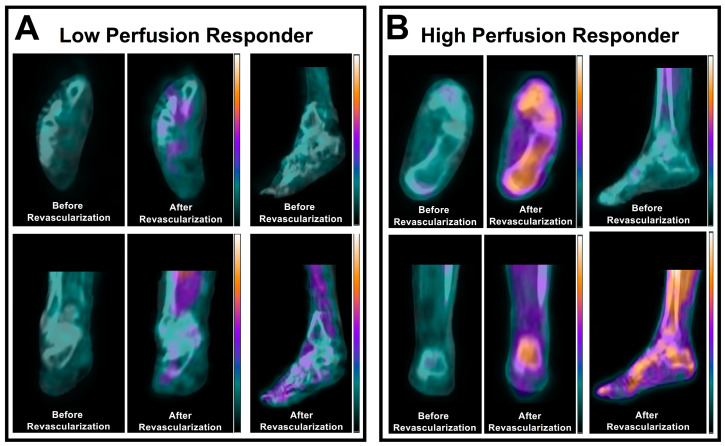
^99m^Tc-tetrofosmin SPECT/CT perfusion imaging before and after endovascular revascularization in (**A**) a low perfusion responder and (**B**) a high perfusion responder with CLTI. The patient with a high perfusion response to revascularization remained amputation-free for one year after intervention, while the low perfusion responder underwent an amputation procedure within one month of intervention [15].

In recent years, hybrid SPECT/CT imaging with ^99m^Tc-tetrofosmin has become increasingly utilized for evaluating regional abnormalities in skeletal muscle perfusion in patients with PAD. A 2018 study by Alvelo et al. [9] evaluated the utility of SPECT/CT imaging for quantifying abnormalities in resting perfusion within 3D vascular runoff territories (i.e., angiosomes) of the foot in patients with chronic limb-threatening ischemia (CLTI). This work demonstrated that SPECT/CT imaging detected significantly lower relative perfusion in the feet of CLTI patients when compared to healthy control subjects [9]. Additionally, this study revealed a mismatch between perfusion values derived from SPECT/CT imaging and the ankle-brachial index, which is a traditional hemodynamic screening test in patients with PAD. These data suggested that SPECT/CT imaging may provide additive assessment of microvascular perfusion in patients with PAD beyond that afforded by conventional PAD screening techniques [9]. Subsequent work from the same research team further demonstrated that ^99m^Tc-tetrofosmin SPECT/CT imaging can be used to quantify serial improvements in regional foot perfusion following lower extremity revascularization in CLTI patients [13], and these SPECT imaging-derived changes in foot perfusion are associated with amputation outcomes in the first 3 and 12 months post-intervention (Figure 1) [15].

Along with evaluating regional foot perfusion and risk for limb loss after peripheral interventions, ^99m^Tc-tetrofosmin SPECT/CT imaging of calf muscle perfusion has also been performed in patients with PAD and demonstrated prognostic value for predicting adverse events. Specifically, Hashimoto et al. [14] utilized SPECT/CT imaging to quantify a lower extremity muscle-to-background ratio (LMBR) as an index of calf muscle perfusion. Patients were then assigned to low and high LMBR perfusion groups, which revealed that a significantly higher number of patients with low calf perfusion experienced major adverse events compared to their high calf perfusion group counterparts.

In addition to characterizing perfusion abnormalities alone, hybrid SPECT/CT imaging has recently demonstrated utility for simultaneously quantifying peripheral artery calcification in patients with PAD during the same imaging session through the use of co-registered CT images. For example, a recent case report using ^99m^Tc-tetrofosmin SPECT/CT imaging in a patient with CLTI and gangrene demonstrated the feasibility of non-invasively detecting local deficits in foot perfusion (via SPECT imaging) while also quantifying peripheral artery calcium burden (via CT imaging). SPECT imaging revealed regions of decreased perfusion at the gangrenous first and second digits as well as at the level of the transmetatarsals, while CT imaging allowed for complementary quantification of peripheral artery calcification in the posterior tibial, peroneal, and anterior tibial arteries. The patient subsequently underwent a transmetatarsal amputation 3 months later that closely aligned with the previously identified anatomical level of perfusion deficit that was identified on SPECT/CT perfusion imaging, suggesting a potential role of nuclear perfusion imaging in detecting viable versus non-viable ischemic tissue and guiding clinicians with amputation/surgical planning [16].

### 2.2. Lower Extremity Osteomyelitis and Infection

PAD patients who present with foot ulcerations, generally due to underlying diabetes mellitus and poor wound healing secondary to arterial insufficiency (ankle brachial index <0.9), may develop soft tissue infections. These infections, if poorly managed, may progress to osteomyelitis, or infection of the underlying bone. Several ^99m^Tc- and gallium-67 (^67^Ga)-based radionuclides have demonstrated utility for targeting and identifying osteomyelitis and tissue infections, which can be critical for reducing adverse events such as amputations by characterizing regions of soft tissue hyperemia and inflammation [55]. These features can be sensitively and specifically identified using radionuclides either alone (^67^Ga) or labeled with inflammatory markers (e.g., ^99m^Tc- or indium-111-labeled white blood cells, WBCs) to accurately identify and differentiate inflammatory from infectious regions of the lower extremities to assist with guiding the medical management of patients at high risk of limb loss [17,56].

Early applications of ^99m^Tc radionuclides for diagnosing PAD-associated osteomyelitis involved two-dimensional imaging with bone scintigraphy; however, the poor specificity and low spatial resolution of scintigraphy proved difficult when differentiating regions of soft tissue versus bone infection [18]. Therefore, 3D SPECT imaging has more recently emerged to overcome the spatial resolution constraints of scintigraphy. Studies comparing the accuracy of SPECT versus scintigraphy for diagnosing osteomyelitis have consistently demonstrated superior capabilities of SPECT imaging. For example, a 2010 study by Heiba et al. [19] evaluated suspected diabetic foot infections using a combined ^99m^Tc hydroxymethlene-diphosphonate (^99m^Tc-HDP) bone scintigraphy and indium-111 (^111^In)-WBC dual-isotope imaging approach and then compared the diagnostic confidence/lesion site detection results of dual-isotope scintigraphic imaging to dual-isotope imaging with SPECT/CT using the same radionuclides. The results of this study revealed that dual-isotope SPECT/CT imaging was the most accurate approach that provided improved detection and discrimination of soft tissue infection and osteomyelitis. Another study by Horger et al. [20] evaluated the use and diagnostic performance of ^99m^Tc-labeled anti-granulocyte antibodies (^99m^Tc-AGA) with two-dimensional scintigraphy versus 3D hybrid SPECT/CT imaging, which demonstrated that hybrid imaging with SPECT/CT improved specificity (89%) compared to scintigraphy (78%) and also reduced the number of false positive results. These studies using ^99m^Tc-labeled tracers highlight the diagnostic abilities and emergence of hybrid imaging modalities for determining the precise anatomical location of osteomyelitis and discriminating osteomyelitis from soft tissue infections in patients with diabetes and PAD.

In addition to ^99m^Tc-labeled imaging approaches, ^67^Ga citrate has been utilized for diagnosing osteomyelitis and differentiating it from non-specific tissue inflammation, though its applications have been more limited than ^99m^Tc. ^67^Ga has also demonstrated lower diagnostic accuracy compared to ^99m^Tc- and ^111^In-labeled tracers traditionally used for scintigraphic or SPECT imaging [21]. To overcome this limitation, ^67^Ga citrate has been previously investigated in conjunction with ^111^In-WBC during scintigraphy and SPECT/CT imaging studies to assess and compare the diagnostic capabilities of these methods for identifying infection [23]. A study by Bar-Shalom et al. [23] found that dual-isotope imaging with SPECT/CT more accurately diagnosed and localized the site of infection and osteomyelitis compared to scintigraphy. Along with using ^67^Ga-citrate for localizing and diagnosing lower extremity osteomyelitis, prior studies have also investigated the utility of ^67^Ga-citrate SPECT/CT imaging for guiding osteomyelitis treatment. A 2013 study by Aslangul et al. [24] specifically coupled ^67^Ga SPECT/CT imaging with percutaneous bone puncture to assist in guiding antibiotic treatment for patients diagnosed with osteomyelitis. This study demonstrated that a combined imaging and bone puncture approach possessed a sensitivity of 88% and specificity of 93.6% for detecting osteomyelitis and subsequently determined that 55% of clinical cases with suspected osteomyelitis did not ultimately necessitate antibiotic treatment [24]. Taken together, hybrid SPECT/CT imaging studies performed in patients with PAD and lower extremity infection have demonstrated potential utility for the diagnosis of osteomyelitis as well as determining appropriate medical management.

## 3. PET/CT Imaging

Although PET scanners are less commonly available and associated with higher relative costs compared to SPECT due to the need for more specialized and expensive radioisotope production facilities (i.e., cyclotrons) and radiochemistry staff, PET imaging generally provides higher sensitivity count detection, improved spatial and temporal resolution imaging, lower levels of ionizing radiation, and accurate quantification of radionuclide uptake in tissues of interest when compared to SPECT imaging [1,12]. PET imaging also allows for the quantitative assessment of a multitude of physiological parameters (e.g., absolute measures of skeletal muscle perfusion and metabolism, peripheral atherosclerosis) that are potentially advantageous in the screening for and diagnosis of lower extremity PAD. The following section highlights the recent developments and applications of PET imaging in the evaluation of patients with PAD.

### 3.1. Skeletal Muscle Perfusion

The first PET-focused investigations of PAD patients in the 1980s by Depairon and colleagues revealed the potential of PET imaging for evaluating skeletal muscle perfusion deficits by demonstrating asymmetrical blood flow and oxygen utilization patterns in the lower extremities of patients with PAD at rest and in response to exercise stress [29,30]. Additional work from the same laboratory later revealed that PET perfusion imaging in PAD patients with bolused H_2_^15^O and ^15^O_2_ allowed for broad physiological assessment of both calf muscle blood flow and oxygen utilization and showed that these PET-derived measures were similar to measures acquired with venous occlusion plethysmography [27]. It was not until 1997 that Burchert and colleagues demonstrated the successful use of ^15^O-water PET imaging to quantify absolute measures of skeletal muscle perfusion in the calf muscles of patients with PAD under various physiological conditions (i.e., rest, exercise stress, pharmacological stress) [28]. More recent work in the 2000s has since demonstrated that PET perfusion measures are also closely correlated with blood flow measures obtained from thermodilution techniques [31] and possess similar diagnostic capabilities as laser Doppler imaging and transcutaneous oxygen pressure in the non-invasive evaluation of calf muscle ischemia in patients with PAD [25]. The test-retest repeatability of ^15^O-water PET/CT imaging was also recently evaluated in healthy subjects in 2024 by Christensen et al. [26], who revealed good repeatability of the method for quantifying skeletal muscle perfusion in various regions of the lower extremities (intraclass correlation coefficients of 0.88 for the calf and 0.87 for the feet).

While traditional PET perfusion radionuclides, such as ^15^O-water, have commonly been used for quantifying skeletal muscle perfusion in the lower extremities, recent research has revealed that 18-fluorine (^18^F)-labeled radionuclides may also possess utility for quantifying skeletal muscle perfusion in the setting of PAD. For example, a 2024 translational study by Chou et al. [47] tested the feasibility of using dynamic PET imaging with 18-fluorine sodium fluoride (^18^F-NaF), a radionuclide traditionally used for quantifying bone perfusion, for quantifying absolute measures of skeletal muscle perfusion in PAD. This dynamic PET imaging approach was first tested and validated in a porcine model of hindlimb ischemia and then translated to patients with varying stages of PAD-related ischemic symptoms. The team further applied the method to a clinical case of peripheral revascularization to assess the feasibility of using this PET imaging approach to quantify perfusion responses to interventions. The study demonstrated that PET-derived measures of perfusion obtained for individual calf muscles were significantly associated with the degree of PAD-related calf symptoms and revealed that PET imaging was capable of quantifying regional perfusion responses to endovascular revascularization in the calf. Thus, this recent work suggests that dynamic PET imaging with commercially available, longer-half-life ^18^F-labeled radionuclides may provide a novel approach for quantifying skeletal muscle perfusion without the need for traditional PET perfusion radioisotopes that possess relatively short half-lives and require more costly on-site production using cyclotrons or generators.

### 3.2. Skeletal Muscle Metabolism and Peripheral Atherosclerosis

In addition to allowing for the investigation of skeletal muscle perfusion in PAD, PET imaging has been applied in recent years to non-invasively quantify ischemia-induced alterations in skeletal muscle metabolism as well as atherosclerotic disease progression associated with PAD. While still relatively understudied in the setting of PAD, PET imaging of skeletal muscle metabolism with ^18^F-fluorodeoxyglucose (FDG), a radiolabeled glucose analog, has demonstrated potential for detecting regional changes in calf muscle metabolism in patients with PAD in a single clinical study [57], thus suggesting that this approach may provide insight into the functional consequences associated with chronic muscle ischemia.

In the assessment of peripheral atherosclerosis, prior studies also utilized ^18^F-FDG as a non-invasive biomarker of atherosclerosis-induced arterial inflammation due to the presence and activation of metabolically active macrophages residing in atherosclerotic plaques that may have an affinity for glucose/^18^F-FDG [35]. In early clinical studies investigating the utility of ^18^F-FDG PET/CT imaging as an indicator of peripheral artery inflammation, Yun et al. [36] demonstrated that arterial retention of ^18^F-FDG was significantly correlated with age. Subsequent work by others similarly confirmed a positive and significant relationship between peripheral artery ^18^F-FDG uptake and patient age [37,38,39]. In addition to aging increasing PET-detectable arterial inflammation, prior studies also revealed higher peripheral artery uptake of ^18^F-FDG on PET images in the presence of cardiovascular risk factors [40], diabetes mellitus-induced arterial stiffness [41], and tobacco use [42]. Aside from correlating strongly with patient-level factors, ^18^F-FDG PET/CT imaging has demonstrated potential as a non-invasive approach for monitoring response to vascular therapies in patients with PAD. Specifically, a 2010 study by Ishii et al. [43] revealed significant reductions in arterial uptake of ^18^F-FDG in response to statin therapy, while a more recent 2021 study by Jiang et al. [33] found that ^18^F-FDG PET/CT imaging was useful for tracking the efficacy of sonodynamic therapy by quantifying serial reductions in arterial inflammation in patients with PAD.

**Figure 2:**
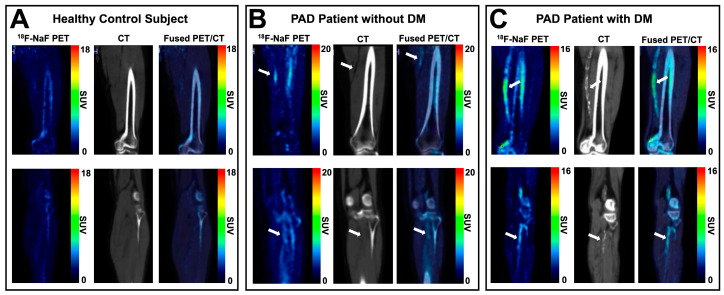
Examples of ^18^F-NaF PET/CT imaging in (**A**) a healthy control subject, (**B**) a PAD patient without diabetes mellitus (DM), and (**C**) a PAD patient with DM, demonstrating increased retention of ^18^F-NaF and increased arterial calcification in lower extremity arteries in the presence of DM and worsening status of PAD [46]. White arrows denote regions of arterial retention of ^18^F-NaF.

Aside from ^18^F-FDG, the utility of ^18^F-NaF as a radionuclide for atherosclerosis-targeted PET/CT imaging in lower extremity PAD has been investigated due to its high affinity for hydroxyapatite, which enables the detection of the active process of vascular microcalcification prior to the development of CT-detectable arterial calcification [58]. ^18^F-NaF PET/CT imaging has been shown to be a feasible approach for investigating atherosclerosis in the lower extremities [48] and has demonstrated a significant association between femoral artery retention of ^18^F-NaF and cardiovascular risk factor profiles of patients with PAD [49,50]. More recent work by Chou et al. [46], who performed vessel-by-vessel analysis of ^18^F-NaF retention on PET/CT images for five major arteries of the lower extremities in patients with PAD, further confirmed that patient-level factors, including the presence of diabetes mellitus and chronic kidney disease, were significantly associated with increasing levels of ^18^F-NaF retention (i.e., active arterial microcalcification) (Figure 2). Importantly, this study also demonstrated that arterial retention of ^18^F-NaF was not significantly associated with CT-detectable calcium burden, thereby indicating that ^18^F-NaF PET/CT indeed detects and differentiates the active process of microcalcification from the established form of vascular calcium in PAD patients. In a follow-up case report by Chou and colleagues [45], ^18^F-NaF PET/CT imaging also revealed potential for predicting the formation and expansion of arterial calcification in multiple lower extremity atherosclerotic lesions in a patient with CLTI, with arterial regions showing elevated ^18^F-NaF at the time of baseline PET/CT imaging subsequently demonstrating increased levels of CT-detectable calcium 18 months later for the same anatomically matched lesion sites. Thus, while still relatively limited in its application to PAD, ^18^F-NaF PET/CT imaging has shown significant potential in the detection and prediction of atherosclerotic progression in the lower extremities of PAD patients and may therefore represent a non-invasive paradigm for monitoring emerging anti-atherogenic therapies for PAD.

## 4. Future Directions and Conclusions

### Angiogenesis

Molecular imaging of angiogenesis, or the formation of new microvasculature, is an emerging topic in the field of PAD that has been explored in the context of chronic ischemic skeletal muscle; however, to date, research involving the use of PET/CT imaging to non-invasively assess the angiogenic process associated with chronic limb ischemia has remained exploratory and pre-clinical in nature. Although there are a multitude of angiogenic mediators that could be targeted for the imaging of angiogenesis, the primary targets that have been utilized to date for pre-clinical PET imaging investigations have been vascular endothelial growth factor (VEGF) and the αvβ3 integrin due to their critical roles in endothelial cell migration and survival [59]. Willmann et al. [60] were one of the first to investigate the utility of copper-64 (^64^Cu)-labeled VEGF_121_ (^64^Cu-DOTA-VEGF_121_) for detecting the angiogenic response to hindlimb ischemia in a rodent model. This initial research work revealed that a peak angiogenic response to limb ischemia was detectable by microPET imaging approximately 1 week after femoral artery occlusion and further demonstrated that the implementation of an exercise training program in rodents significantly increased PET-detectable angiogenesis within ischemic hindlimbs. Additional PET imaging investigations focused on targeting the αvβ3 integrin have traditionally accomplished this feat via radiolabeling of the cyclic arginine-glycine-aspartate (RGD) peptide, which has been shown to possess a high affinity for the αvβ3 integrin [59]. Examples of such PET radionuclides that have been developed in the last 15 years using the RGD peptide are ^68^Ga-NOTA-RGD [61] and ^64^Cu-NOTA-PEG4-cRGD2 [62], with the latter demonstrating potential for non-invasively tracking therapeutic responses to stem cell therapy in a rodent model of hindlimb ischemia. Along with PET imaging of VEGF and αvβ3 integrins, Orbay et al. [63] demonstrated the utility of targeting CD105 (i.e., endoglin), which is an overexpressed protein on newly formed blood vessels, through the use of ^64^Cu-NOTA-TRC105 microPET imaging. Their initial investigation using this radionuclide revealed that serial microPET imaging in a mouse model of hindlimb ischemia detected a peak angiogenic response to femoral artery occlusion at 3 days post-occlusion. Later work from the same research team using ^64^Cu-NOTA-TRC105 also demonstrated that microPET imaging was capable of detecting angiogenic responses to pravastatin administration in a rodent model of hindlimb ischemia [64]. Compared to perfusion- and atherosclerosis-targeted imaging studies in PAD, non-invasive monitoring of angiogenesis has remained pre-clinical in nature and relatively understudied. However, continued development of angiogenesis-targeted PET radionuclides may provide a novel platform and offer valuable insight into therapeutic responses for emerging treatments and regenerative medicine approaches in PAD patients in the future.

Non-invasive imaging of PAD is increasingly moving towards hybrid imaging modalities that simultaneously allow for the assessment of the functional and structural consequences of this disease, which may enhance the future detection of and risk stratification for PAD, improve the identification of physiological processes associated with therapeutic efficacy, and open the door for guiding personalized medicine in this clinical population. In recent years, there has been a surge of artificial intelligence (AI)-based image analysis and predictive modeling techniques utilized for patients with PAD [65] that may offer continued opportunities for research endeavors given the emerging role of predictive modeling in coronary artery disease (CAD) within the past decade [66]. From an image analysis standpoint, deep learning has recently been applied in clinical PAD datasets to automate the analysis of relevant imaging data, including Doppler ultrasound waveforms [67], CT angiography images [68], and magnetic resonance perfusion images acquired in PAD patients [69], and i5 has consistently demonstrated the ability to accelerate the processing and interpretation of clinical images. Additionally, in recent years, models such as random forest models have shown excellent sensitivity and specificity for diagnosing PAD based on common PAD-associated comorbidities [70,71]. Additional recent proof-of-concept work in PAD using a long short-term memory network in combination with logistic regression and support vector machines has similarly demonstrated high specificity and sensitivity in the diagnosis of PAD based on features extracted from peripheral artery Doppler waveforms [72]. Future application of machine learning models to nuclear medicine may allow for automated regional analysis of peripheral skeletal muscle for perfusion analysis and/or lesion- or vessel-based analysis of atherosclerosis, which could provide efficient detection and quantification of PAD pathophysiology, thereby expediting the diagnosis, determination of disease severity, and implementation of appropriate patient-specific interventions or therapies. While the applications of nuclear imaging have traditionally been limited in PAD, emerging clinical studies continue to reveal the potential role of nuclear medicine techniques for improving our understanding of PAD pathophysiology and creating future opportunities for the early detection and serial monitoring of PAD patients.

## Figures and Tables

**Table 1:** Clinical applications of SPECT and PET imaging for evaluation of PAD.

Modality	Radioisotope	Physiologic Target	Reference
SPECT	^201^Tl	perfusion	[1,8,9,10,11,12]
	^99m^Tc-sestamibi	perfusion	[1,11,12]
	^99m^Tc-tetrofosmin	perfusion	[1,9,11,12,13,14,15,16]
	^99m^Tc-diphosphonate + ^111^In-WBC	osteomyelitis, infection	[17,18,19]
	^99m^Tc-antigranulocyte antibodies	osteomyelitis, infection	[20]
	^67^Ga-citrate	osteomyelitis, infection	[21,22,23,24]
PET	H_2_^15^O	perfusion	[25,26,27,28,29,30,31]
	^15^O_2_	perfusion	[27,29,30]
	C^15^O_2_	perfusion	[29,30]
	^18^F-FDG	metabolism, atherosclerosis	[32,33,34,35,36,37,38,39,40,41,42,43]
	^18^F-NaF	perfusion, atherosclerosis	[34,44,45,46,47,48,49,50]
	^11^C-acetate	atherosclerosis	[51]

## Data Availability

No new data were created or analyzed in this study. Data sharing is not applicable to this article.
